# Competence-Independent Activity of Pneumococcal Enda Mediates Degradation of Extracellular DNA and Nets and Is Important for Virulence

**DOI:** 10.1371/journal.pone.0070363

**Published:** 2013-07-31

**Authors:** Luchang Zhu, Zhizhou Kuang, Brenda A. Wilson, Gee W. Lau

**Affiliations:** 1 Department of Pathobiology, University of Illinois at Urbana-Champaign, Urbana, Illinois, United States of America; 2 Department of Microbiology, University of Illinois at Urbana-Champaign, Urbana, Illinois, United States of America; University of Tübingen, Germany

## Abstract

Membrane surface localized endonuclease EndA of the pulmonary pathogen *Streptococcus pneumoniae* (pneumococcus) is required for both genetic transformation and virulence. Pneumococcus expresses EndA during growth. However, it has been reported that EndA has no access to external DNA when pneumococcal cells are not competent for genetic transformation, and thus, unable to degrade extracellular DNA. Here, by using both biochemical and genetic methods, we demonstrate the existence of EndA-mediated nucleolytic activity independent of the competence state of pneumococcal cells. Pneumococcal mutants that are genetically deficient in competence development and genetic transformation have extracellular nuclease activity comparable to their parental wild type, including their ability to degrade neutrophil extracellular traps (NETs). The autolysis deficient Δ*lytA* mutant and its isogenic choline-treated parental wild-type strain D39 degrade extracellular DNA readily, suggesting that partial cell autolysis is not required for DNA degradation. We show that EndA molecules are secreted into the culture medium during the growth of pneumococcal cells, and contribute substantially to competence-independent nucleolytic activity. The competence-independent activity of EndA is responsible for the rapid degradation of DNA and NETs, and is required for the full virulence of *Streptococcus pneumoniae* during lung infection.

## Introduction

EndA is reported to be a membrane-localized pneumococcal endonuclease [1]. It was first implicated to play a role in genetic transformation by Kohoutova [2], and subsequently confirmed by Lacks and colleagues [3]–[5]. During genetic transformation, EndA degrades one strand of double stranded DNA (dsDNA) and converts it into single stranded DNA (ssDNA) for uptake and recombination [6]. Acid soluble DNA fragments or nucleotides generated during DNA degradation are released into the culture medium [4], and are only detectable during competence development [7]. These observations were further confirmed by Berge and colleagues [8]. They showed that the Δ*comD* mutant, which lacks the histidine kinase receptor required for the induction of the competence regulon and genetic transformation, is both unable to develop competence and degrade DNA. In addition, the *comEA* and *cglABCDEFG* operons, which encode the apparatus for DNA binding and uptake, are required for EndA-mediated DNA degradation. These authors proposed that DNA attaches to the apparatus in competent pneumococcal cells, allowing the membrane-localized EndA to gain access to the extracellular DNA. For pneumococcal cells not under the competent state, EndA is unable to gain access to donor DNA, and is incapable of degrading extracellular DNA.

Demonstration of EndA’s role in pneumococcal virulence is comparatively recent. An EndA-deficient mutant was identified in a signature-tagged mutagenesis screen in mouse lungs [9]. Furthermore, EndA degrades neutrophil extracellular traps (NETs) [10]. Composed of both DNA and antibacterial proteins, NETs are released by activated neutrophils to capture and kill bacterial cells [11]. Thus, it is conceivable that rapid degradation of NETs by EndA releases captured pneumococcal cells and facilitates their dissemination.

In this study, we present experimental evidence that majority of the nucleolytic activities of EndA are independent of competence development, and the “competence-independent activity” of EndA contributes to lung infection. In addition, EndA is secreted during pneumococcal growth.

## Materials and Methods

### Synthetic CSP1, Bacterial Strains and Growth Conditions

CSP1 (≥95% purity) was synthesized by Elim Biopharm. *S. pneumoniae* strains are listed on Table 1. Wild-type strain D39 [12] was a gift from Dr. David Briles (University of Alabama-Birmingham). R6 is a capsule-deficient mutant derived from D39 [13] whereas 0100993 is a highly encapsulated serotype III clinical isolate [14]. Mutant strains Δ*comD*, Δ*comA,* Δ*cglABCDEFG,* Δ*lytA,* and Δ*endA* were generated by nonpolar deletions in D39 using the Janus cassette as previously described [15]. Strain JC0923, which carries both an insertion of the *lacZ* gene under the control of the *comX* promoter and a nonpolar deletion in the *comA* gene, was generated by transforming Δ*comA* with the genomic DNA from D39pcbpD::lacZ [16], [17]. Bacteria were streaked from frozen stocks onto THB (Todd Hewitt Broth) agar containing 5% defibrinated horse blood at 37°C with 5% CO_2_. Fresh colonies were grown in THB or CTM (complete transformation medium) [18] to desired density.

**Table 1:** *S. pneumoniae* strains used in this study.

Strains	Relevant characteristics	Reference
D39	Wild-type	[12]
AD2064 (Δ*comCDE*)	A competent deficient D39 derivative generated by deleting the *comCDE* operon using the Janus cassette	This work
AD1863 (Δ*cglABCDEFG*)	A transformation deficient D39 derivative generated by deleting the *cglABCDEFG* operon using theJanus cassette	This work
AD1737 (Δ*lytA*)	A D39 derivative deficient in major autolysin generated by deleting the *lytA* gene usingthe Janus cassette	This work
AD1762 (Δ*endA*)	A transformation deficient D39 derivative generated by deleting the *endA* gene using the Janus cassette	This work
AD0049 (Δ*comA*)	A D39 derivative deficient in spontaneous competence generated by deleting the *comA* gene usingthe Janus cassette	This work
IN1643 (D39pcomX::lacZ)	D39 with a promoterless *lacZ* reporter gene fused behind the *comX* promoter	This work
JC0923	D39pcomX::lacZ strain with a deletion in the *comA* gene	This work
CPM3	R6 with a promoterless *lacZ* reporter gene fused behind the *comX* promoter	[31]
CP1296	R6 strain with a modified *rpsL* gene that confers resistance to streptomycin	[15]
R6	A capsule-deficient derivative of D39	[13]
AR2042 (R6Δ*comD*)	A competence-deficient R6 derivative generated by deletion of the *comD* gene using the Janus cassette	This work
AR1779 (R6Δ*endA*)	A R6 derivative generated by the deletion of the *endA* gene using the Janus cassette	This work
TIGR4	Wild-type	[35]
AT2236 (TIGR4Δ*comD*)	A competence-deficient TIGR4 derivative generated by deleting the *comD* using the Janus cassette	This work
AT1762 (TIGR4Δ*endA*)	A transformation-deficient TIGR4 derivative generated by deleting the *endA* gene using the Janus cassette	This work
0100993	Serotype 3 clinical isolate	[14]
ST2012 (0100993Δ*cap3A*)	A capsule-deficient 0100993 derivative generated by deleting the *cap3A* gene using the Janus cassette	This work
AG2236 (0100993Δ*comD*)	A competence-deficient 0100993 derivative generated by deleting the *comD* gene using the Janus cassette	This work
AG1762 (0100993Δ*endA*)	A transformation-deficient 0100993 derivative generated by deleting the *endA* gene using theJanus cassette	This work

### DNA Degradation Assay

Pneumococcal cells were grown in THB to early log phase around 10^8^ CFU/ml (1 ml). The OD 600 nm at this bacterial concentration for D39, R6 and 0100993 were 0.15, 0.1 and 0.4, respectively. The differing OD600nm at 10^8^ CFU/ml between strains D39 and 0100993 is due to the presence of different capsules. Pneumococcal cells were washed three times with PBS and resuspended in 1 ml of fresh THB and added to column purified PCR products of the streptomycin resistance *rpsL* gene (30 µg). At indicated time points, a 100 µl aliquot of the bacteria-DNA mixture was withdrawn and immediately centrifuged at 12000 rpm for 2 min. DNA-containing supernatants were stored in −80°C to prevent further degradation. The integrity of DNA was visualized by agarose gel electrophoresis. For degradation of DNA in the CTM medium**,** pneumococcal strains were grown in THB to 10^8^ CFU/ml (1 ml), washed three times with PBS and resuspended in 1 ml of CTM. In addition, we also grew pneumococcal strains in CTM to 10^8^ CFU/ml (1 ml), washed, and resuspended in 1 ml of THB. Degradation of DNA was performed as above. As controls, DNA degradation was inhibited using the nuclear inhibitor aurintricarboxylic acid (ATA) (15 µg/ml). ATA inhibits the enzymatic activities of most nuclei acid binding proteins [19]. For the dsDNA integrity assay, supernatant that contained donor DNA was stained with ethidium bromide (0.5 µg/ml). The fluorescence was measured using a fluorometer at indicated wavelengths (excitation: 300 nm; emission: 600 nm). To assay DNA degradation on agar plates, pneumococcal strains were streaked on THB agar incorporated with 166 µg/ml salmon sperm DNA. After 12 hr, DNA plates were flooded with 5 ml of 1 N HCl as previously described [19]. DNA degradation appeared as transparent zones of clearance.

### Degradation of [α32P]-dATP-labeled DNA

P-32 labeled donor DNA was amplified by PCR using 1x PCR master mix (Thermoscientific) and D39 chromosomal DNA (100 µl reaction size). PCR was carried out with the *rpsL* primers in the presence [α32P]-dATP (Amersham; specific activity 111×10^6^ kBq mmol−1) for 40 cycles with the following settings: 30 seconds at 94°C, 30 seconds at 54°C, 60 seconds at 72°C, followed by a 10 min extension step. Hot PCR products were purified by QIAquick PCR Purification Kit (Qiagen). Hot DNA (5 µCi) was exposed to 10^8^ CFU/ml Δ*comA* cells (in 1 ml) with or without CSP1 stimulation. The Δ*endA* cells (10^8^ CFU/ml) and THB were used as controls. To determine the extent of DNA degradation, an aliquot of 100 µl was withdrawn from each group at indicated time points after incubation. The samples were centrifuged at 12000 rpm for 1 min to remove bacterial cells. Large intact hot DNA fragments within the supernatant were removed by QIAquick PCR Purification columns. The filtrates (20 µl), which contained the hot nucleotides and small DNA fragments (<100 bp), were spotted on filter paper, dried, and exposed in a phosphoimager exposure cassette for 8 hr and scanned with the FujiFilm FLA-3000 PhosphorImager. Phosphoimager signals were quantified by Image J. For quantitative determination of released nucleotides/small DNA fragments, the filtrates (20 µl), which contained the hot nucleotides and small DNA fragments (<100 bp), were mixed thoroughly with 5 ml of Cytoscint* Scintillation Cocktail (Thermoscientific). Radioactivity was quantified by using a Tri-Carb® 2100TR Liquid Scintillation Counter (Perkin Elmer).

### Analysis of the EndA Secretion

Pneumococcal strains TIGR4, 0100993, and D39 and its derivatives *ΔcomD* and *ΔlytA,* were cultured in THB to ∼ 10^8^ CFU/ml. One ml of culture was withdrawn and subjected to centrifugation. Bacterial cells were then washed three times with PBS to remove cultural medium and resuspended into 1 ml of fresh THB. Resuspended bacterial strains were allowed to grow in the 37°C incubator supplemented with 5% CO_2_. At 0, 30, 60 and 90 min, supernatant samples of each bacterial culture were collected. Pneumococcal cells were removed using the 0.25 µm filters. 20 µl of bacterial cells or supernatant was boiled with 5 µl of 5x SDS loading buffer (0.25% Bromophenol blue, 0.5 M dithiothreitol, 50% Glycerol, 10% SDS, 0.25 M Tris-Cl, pH 6.8) for 5 min, and subjected to SDS-PAGE in an acrylamide gel incorporated with 15 µg/ml of salmon sperm DNA. Following electrophoresis, the gel was washed with pure water to remove SDS, allowing renaturation of proteins. After 12 hr of incubation in 2 mM MgCl_2_ solution at 37°C, the gel was stained with 10 µg/ml of ethidium bromide to visualize bands of DNA clearance caused by pneumococcal nuclease. A band of clearance at 25 kDa indicates the presence of EndA nucleolytic activities.

To further compare the amount of EndA secreted by pneumococcus growing in THB versus in CTM, washed D39 cells (10^8^ CFU/ml) were resuspended into 1 ml of fresh THB or CTM, respectively, allowed to grow in the 37°C incubator supplemented with 5% CO_2_ for 2 hr. One ml of cell-free supernatant from D39 cells growing in THB were diluted 10, 100 and 1000 folds. Nuclease activity of different diluents was compared against cell-free supernatant from D39 cells grown in CTM. Each supernatant sample was incubated with hot PCR product at 37°C for 1 hr. The release of nucleotides/small DNA fragments was quantified by using the Tri-Carb® 2100TR Liquid Scintillation Counter (Perkin Elmer).

### Genetic Transformation Assay

Pneumococcal JC0923 cells were grown to OD 600 nm 0.15 in THB (pH 6.8), washed and resuspended in fresh THB (pH 8.3) [14], or CTM, and stimulated with 400 ng/ml CSP1. Donor DNA was added to the final concentration of 30 µg/ml. Donor DNA was generated by amplifying a mutated *rpsL* gene and its flanking regions from a streptomycin resistant strain CP1296 (Table 1), using the following primers: *rpsL* upper 5′-GGGCTAGTAGAAGTAGTTGG-3′; *rpsL* lower: 5′-CGGAAGTGTGCGAATGCACG-3′ (PCR product size: 1633 bp). The transformation mix was then incubated at 37°C with 5% CO_2_ for 2 hr. Transformants were selected on THB agar supplemented with 100 µg/ml streptomycin after serial dilutions.

### Activation Assay of *comX* and Promoter

JC0923 cells were grown in THB (pH 6.8) until OD 600 nm of 0.15, washed and resuspended in fresh THB (pH 8.3) or CTM. CSP1 (400 ng/ml) was added to the bacterial culture and incubated at 37°C for 30 min. β-galactosidase activity was measured according to previously published protocols [20] and expressed as Miller units.

### Neutrophil NETs Degradation Assay

Human neutrophils (Innovative Research) grown in RPMI medium were exposed to 10 mM H_2_O_2_ in 37°C for 20 min to induce the formation of NETs [21]. Activated neutrophils were incubated for 60 min with RPMI, 10^8^ cells of D39, Δ*comCDE*, Δ*cglABCDEFG*, or Δ*endA*, respectively. Neutrophils were fixed with 4% paraformaldehyde and stained with DAPI to visualize NETs degradation by using a fluorescence microscope (Zeiss LSM 700).

### Ethics Statement

The animal study was carried out in strict accordance with the recommendations in the Guide for the Care and Use of Laboratory Animals of the National Institutes of Health. The protocol was approved by the Institutional Animal Care and Use Committee (IACUC) at the University of Illinois at Urbana-Champaign (Protocol Number: 12230).

### Mouse Acute Pneumonia Infection

CD1 mice (6-week old, n = 10) (Charles River) were housed in positively-ventilated microisolator cages with automatic recirculating water, located in a room with laminar, high efficiency particle accumulation–filtered air. The animals received autoclaved food, water, and bedding. Mice were anesthetized with isoflurane and intranasally administered 10^6^ CFU of D39, Δ*cglABCDEFG*, or Δ*endA* cells. The infected mice were monitored for 48 hr before the lungs were harvested for bacterial enumeration. Moribund animals that displayed rough hair coat, hunched posture, distended abdomen, lethargy or inability to eat or drink were euthanized. Animal studies were carried out in strict accordance to the protocol (#12230) approved by the IACUC at the University of Illinois at Urbana-Champaign.

### Statistical Analyses

Statistical analyses of *in vitro* experiments were performed using the Student’s *t*-test and one-way analyses of variance (ANOVA). Statistical significance of bacterial burden in mouse lungs was compared using the GraphPad Prism statistical software package. A significant difference was considered to be *p*<0.05.

## Results

### Degradation of Extracellular DNA by EndA does not Require Components of the Competence Regulon

**Figure 1:**
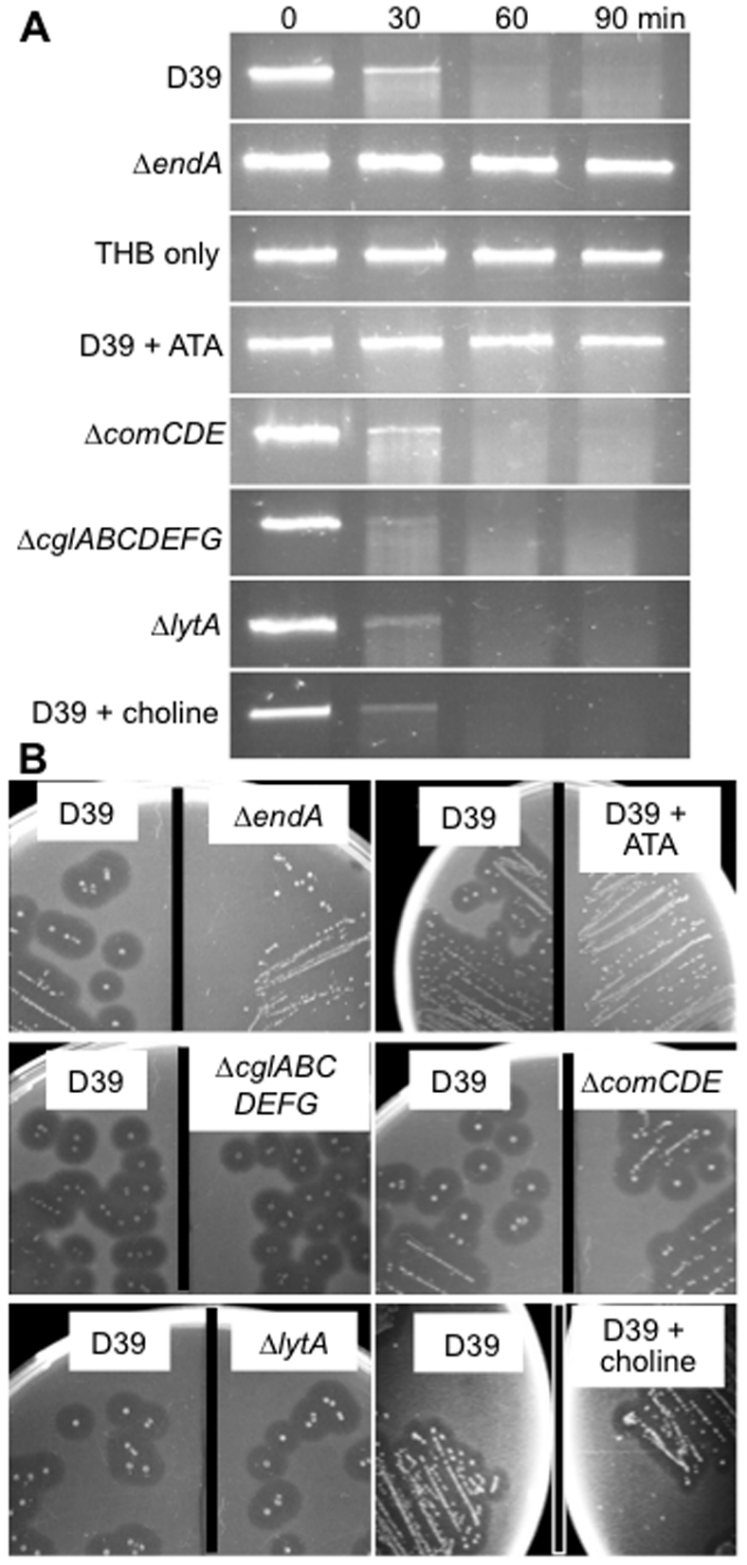
Extracellular DNA degradation by pneumococcus EndA is independent of the competence regulon and cell autolysis. (A) The *rpsL* PCR products (30 µg/ml) were exposed to the wild-type D39 and isogenic mutants for the indicated time intervals, or in the presence or absence of ATA or 2% choline chloride. The integrity of DNA was visualized by agarose gel electrophoresis. (B) Competence-deficient pneumococcal mutants degrade salmon sperm DNA efficiently. Pneumococcal cells were streaked onto the THB agar supplemented with salmon sperm DNA in the presence or absence of ATA or choline chloride. After 24 hr, DNA degradation was visualized by flooding the plates with HCl. Three independent experiments were performed for both A and B with similar results. The data from one typical experiment are shown.

We compared the ability of the wild-type pneumococcal strain D39 and its isogenic endonuclease-deficient Δ*endA* mutant to degrade extracellular DNA. DNA was rapidly degraded by D39 (Fig. 1A). After 60 min of incubation with D39 cells, no clear DNA band was visible. In contrast, the DNA exposed to Δ*endA* remained intact for the entire duration of 90 min, similar to the DNA exposed to THB (Fig. 1A). These results were confirmed by degradation of salmon sperm DNA incorporated into agar plates (Fig. 1B). Transparent halos surrounded D39 colonies, indicating DNA was degraded. In contrast, no halos were visible on the Δ*endA* plates. The nuclease inhibitor aurintricarboxylic acid (ATA) (15 µg/ml) [22] inhibited EndA activities, with no significant amounts of DNA degradation detected during the 90 min incubation (Fig. 1A). ATA also inhibited the degradation of salmon sperm DNA (Fig. 1B). Collectively, these results indicate that EndA is the principle endonuclease that rapidly degrades extracellular DNA in pneumococcus.

To determine the relationship between competence development and nucleolytic activity of EndA, we compared the DNA degradation ability of D39 versus its isogenic mutants Δ*comCDE* and Δ*cglABCDEFG*. The Δ*comCDE* mutant is deleted in the operon encoding the 17 amino acid peptide pheromone competence stimulating peptide 1 (CSP1), the histidine kinase receptor ComD, and the response regulator ComE, respectively, which together, regulates the competence regulon [23]. As such, this mutant is unable to enter the competent state [23]. The Δ*cglABCDEFG* is deficient in the DNA uptake apparatus required for initial DNA recruitment and binding [8]. Both Δ*comCDE* and Δ*cglABCDEFG* mutants degraded extracellular DNA as efficiently as D39 (Fig. 1A–B). Collectively, these results suggest the existence of EndA activity that is independent of the competence development in pneumococcus.

### DNA Degradation by EndA is not due to Pneumococcal Autolysis

Autolysis of pneumococcus during growth is primarily mediated by the autolysin LytA [24], and may release EndA from cell membrane or cytoplasm into the environment. However, the Δ*lytA* mutant degraded extracellular DNA as efficiently as D39 (Fig. 1A–B). In addition, D39 cells treated with 2% choline chloride – which inhibit the autolytic activities of all pneumococcal lytic proteins LytA, LytB, LytC and CbpD [25]–[29] – degraded extracellular DNA as efficiently as untreated D39 cells (Fig. 1A–B). These results indicate that pneumococcal autolysis does not contribute significantly to the competence-independent EndA activity.

### Induction of Competence Contributes Minimally to the Overall Amount of DNA Degradation by Pneumococcus

**Figure 2:**
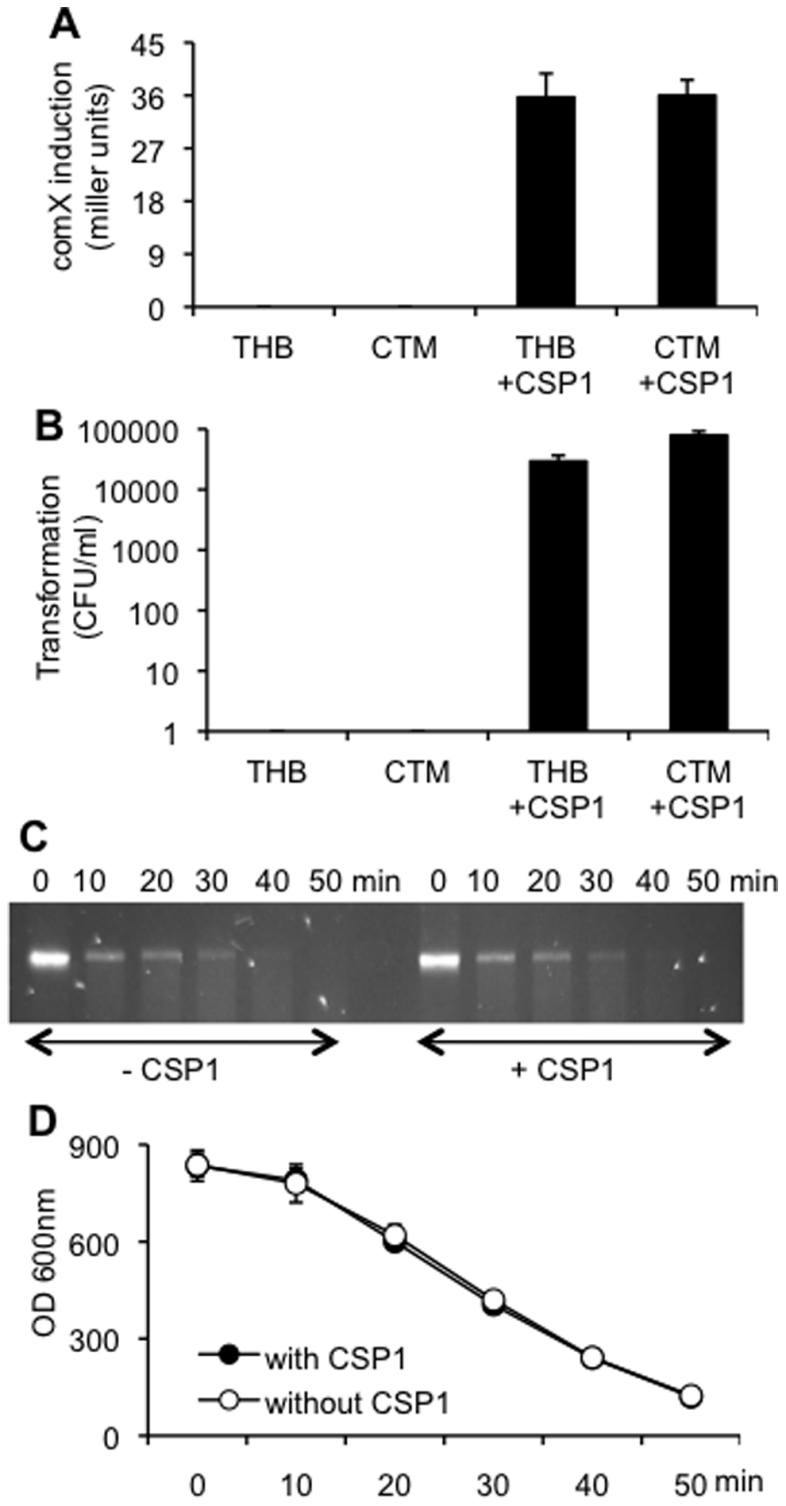
Induction of competence with CSP1 does not appreciably increase EndA-mediated DNA degradation. (A–B) Induction of both *comX* expression (A) and genetic transformation (B) in the JC0923 cells grown in THB or CTM with or without exposure to 400 ng/ml CSP1. Genetic transformation was performed with the addition of 30 ug/ml *rpsL* PCR products. Experiments were performed in triplicates and repeated three times. The means ± SD of one typical experiment are shown. (C) Integrity of the *rpsL* DNA exposed to THB-grown JC0923 cells in the presence or absence of CSP1. (D) Measurement of dsDNA integrity (from C) with ethidium bromide (excitation: 300 nm; emission: 600 nm). Experiments were performed in triplicates and repeated three times. The means ± SD of one typical experiment are shown.

To determine if the extracellular DNA degradation by EndA was dependent on the development of competence, we examined the induction of competence by using the pneumococcal strain JC0923 (Table 1) in the presence or absence of CSP1. JC0923 cells carry a *lacZ* reporter gene under control of the *comX* promoter and a deletion of the *comA* gene. ComX is a competence specific sigma factor that positively regulates the transcription of genes for DNA uptake and recombination [30], [31]. ComA is an ABC transporter that exports CSP1 [32]. Therefore, JC0923 serves as an ideal test strain to monitor *comX* expression and genetic transformation, which could only be triggered by exogenously supplied CSP1. We compared the transformation efficiency of JC0923 in THB and in CTM, a medium that allows high transformation efficiency [18]. As shown in Fig. 2A and 2B, the expression of *comX* promoter driven LacZ is comparable in both media, suggesting that the induction of competence by CSP1 in THB was successful. Nevertheless, transformation efficiency in the CTM is ∼ 3 times higher than in the THB. JC0923 cells grown in THB were able to degrade the *rpsL* DNA with equal efficiency in the presence or absence of exogenously supplied CSP1 (Fig. 2C). DNA staining with ethidium bromide over the time course of the experiment showed that the kinetics of DNA degradation was indistinguishable between the JC0923 cells with or without CSP1 treatment (Fig. 2D). Collectively, these results suggest that the contribution of competence induction to the overall DNA degradation by pneumococcus is negligible.

### Competence-independent EndA Nucleolytic Activity is Conserved in Different Pneumococcal Strains and is Culture Medium Dependent

**Figure 3:**
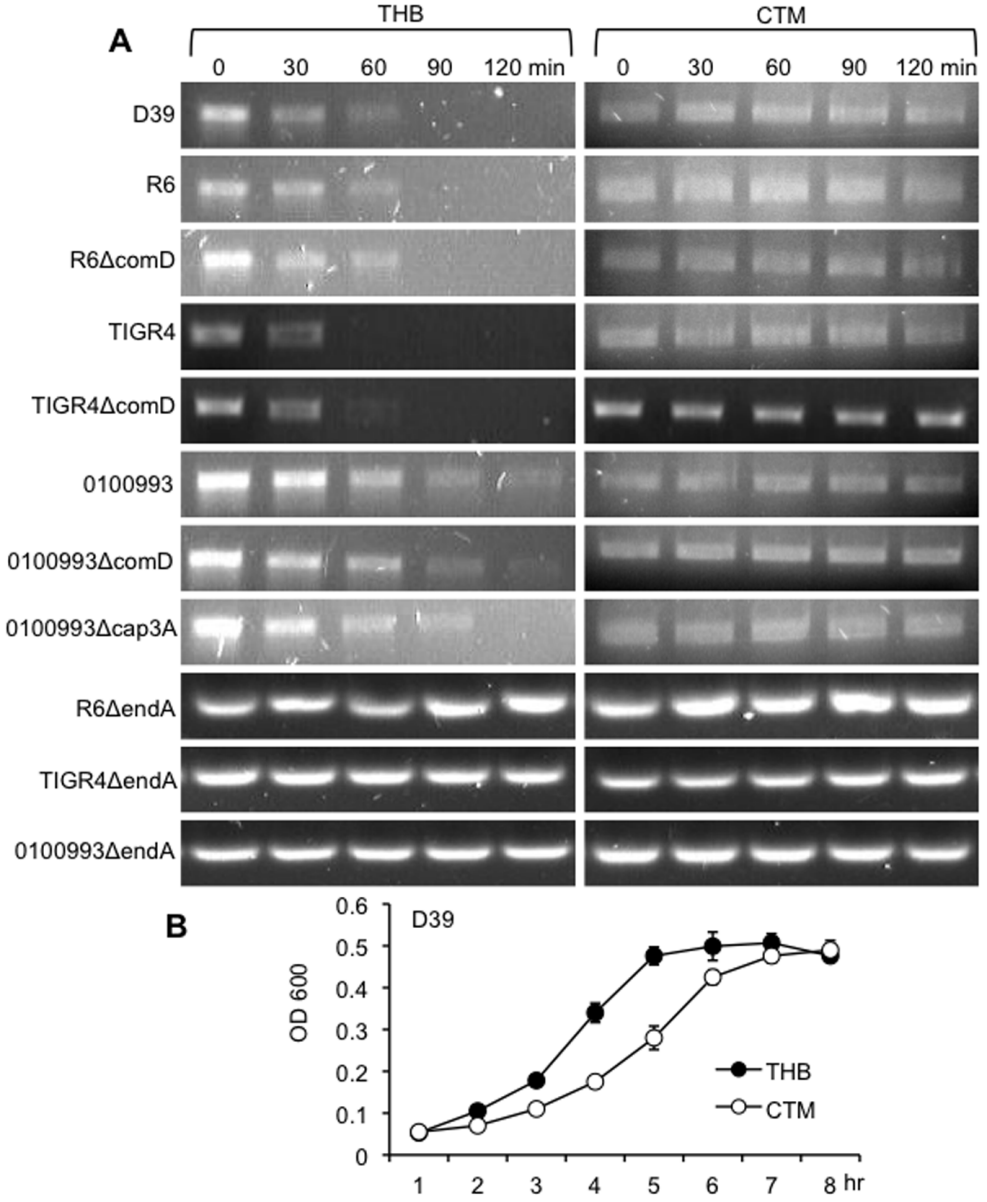
EndA-mediated competence-independent DNA degradation is conserved in multiple pneumococcal strains and is growth medium-dependent. (A) Pneumococcal strains D39, R6, 0100993, Tigr4 and their isogenic derivatives were cultured in THB to 10^8^/ml concentration, washed and resuspended in fresh THB or CTM. The *rpsL* PCR products (30 µg/ml) were exposed to the pneumococcal strains for the indicated time intervals. The integrity of DNA was visualized by agarose gel electrophoresis. The experiments were repeated independently three times. The results from one typical experiment are shown. (B) Growth kinetics of D39 in THB versus CTM. The experiments were performed independently in triplicates and repeated three times. The means ± SD of one representative experiment are shown.

To determine if the competence-independent activity of EndA is a wide spread phenomenon, we tested the pneumococcal strains R6, 0100993, and TIGR4 as well as their isogenic Δ*comD* mutants for their ability to degrade extracellular DNA. D39 was used as positive control for DNA degradation. Pneumococcal strains were grew in THB, washed and resuspended in THB or CTM medium and examined for DNA degradation. As shown in Fig. 3A, R6, 0100993 and TIGR4 possess different nucleolytic activity levels. In contrast, their isogenic Δ*endA* mutants are unable to degrade DNA. Importantly, the Δ*comD* mutants degrade DNA as efficiently as their respective parental strains R6, 0100993 and TIGR4, confirming that the competence system is not important for EndA-mediated DNA degradation. In addition, D39 and its unencapsulated derivative R6, as well as 0100993 and its capsule-deficient Δ*cap3A* mutant, show similar amount of DNA degradation activities. These results suggest capsule does not influence EndA activity in these strains. However, the nucleolytic activity appears to be influenced by pneumococcal growth. D39 grew at slower rate in the CTM (Fig. 3B). Similarly, TIGR4 and 0100993 also grew slower in CTM (data not shown). Degradation of extracellular DNA was severely impaired when D39 was grown in CTM (Fig. 3A).

**Figure 4:**
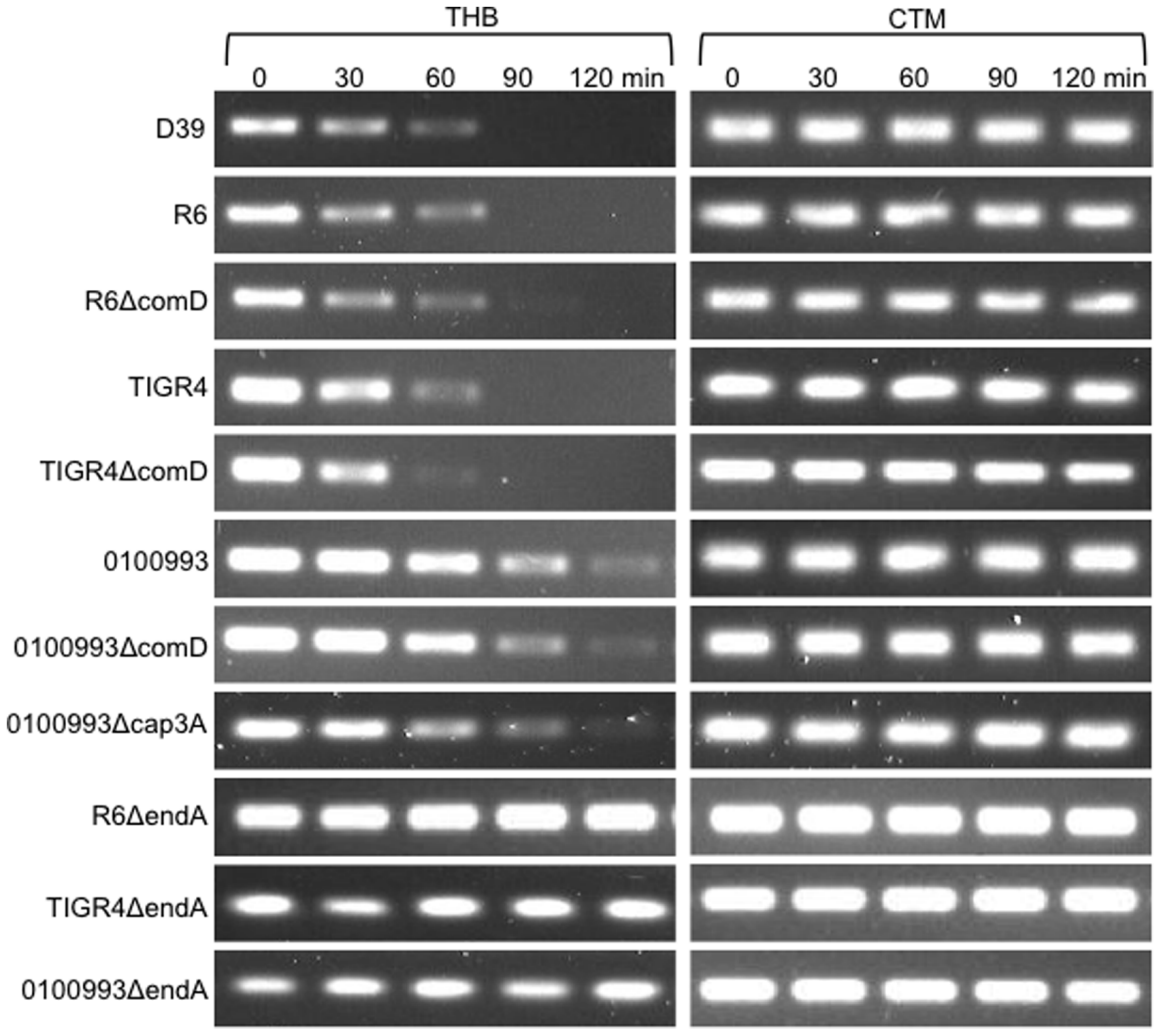
Pneumococcal strains cultured in CTM have impaired ability to degrade DNA. Pneumococcal strains D39, R6, 0100993, Tigr4 and their isogenic derivatives were grown in CTM to 10^8^/ml concentration, washed and resuspended in fresh THB or CTM. The *rpsL* PCR products (30 µg/ml) were exposed to the pneumococcal cells for the indicated time intervals. The integrity of DNA was visualized by agarose gel electrophoresis. The experiments were repeated independently three times. The results from one typical experiment are shown.

Because pneumococcal strains were cultured in THB before been resuspended in the nutritionally poorer CTM for DNA degradation, there is a possibility that the impaired DNA degradation in CTM was due to the inability of pneumococcus to adjust to poorer growth conditions. To rule our this possibility, pneumococcal strains were also cultured in CTM to achieve 10^8^ CFU/ml, washed and resuspended in THB or CTM medium and examined for DNA degradation. Again, D39, R6, TIGR4, 0100993 and their competence-deficient *ΔcomD* derivatives degraded DNA efficiently in THB, but not in CTM (Fig. 4). These results suggest that EndA-mediated degradation of extracellular DNA is partially dependent on the nutritional condition of growth medium. Collectively, the aforementioned results suggest that competence-independent nucleolytic activity of EndA is conserved in different pneumococcal strains. Furthermore, EndA degrades DNA with equal efficiency in the presence or absence of capsule. However, a richer medium such as THB allows better bacterial growth and higher nucleolytic activities.

### Competence-dependent EndA Activity Mediates Rapid Release of Small DNA Fragments Right After CSP1 Stimulation

**Figure 5:**
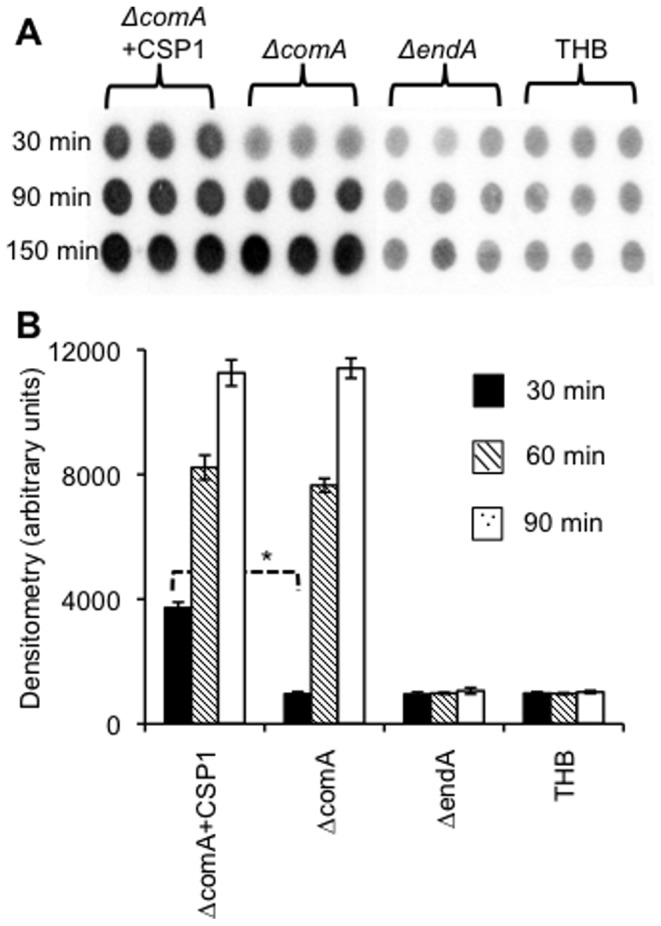
Nucleolytic activity of EndA during competence induction by CSP1. PCR-amplified P-32 labeled donor DNA (5 μ-3) was exposed to Δ*comA* cells in the presence or absence of 400 ng/ml CSP1. The Δ*endA* cells and THB were used as controls. Experiments were performed in triplicates. (A) Scanned image of small hot DNA fragments (<100 bp) and nucleotides spotted on filter paper and exposed to a phosphoimager cassette. (B) Quantification of dots (n = 3) in A. *p<0.05 when comparing the densitometry number of *ΔcomA* supplemented with CSP1 against *ΔcomA* alone at 30 min and 90 min post CSP1 exposure.

Contrary to previous reports [7], [8], our results indicate that DNA degradation by EndA is not dependent on the development of pneumococcal competence. To determine if the discrepancy is caused by different methods of measuring nuclease activity, we examined DNA degradation mediated by competent and non-competent pneumococcal cells by measuring the EndA-mediated release of small DNA fragments and nucleotides, as previously published [7], [8]. Degradation of P-32-labeled hot DNA was compared among Δ*comA* cells with or without CSP1 stimulation, Δ*endA* cells or THB control. As expected, substantial release of small DNA fragments and nucleotides was only observed in the Δ*comA* and Δ*comA*+CSP1 groups (Fig. 5A–B), indicating that EndA was responsible for DNA degradation. However, the initial rate of DNA degradation differed significantly. After 30 min of incubation, the amount of small DNA fragments and nucleotides released by CSP1-stimulated competent Δ*comA* cells was 3.9 fold higher than untreated Δ*comA*, as determined by densitometry analysis. In contrast, the amount of small DNA fragments and nucleotides released by the Δ*comA* group at this time point was only slightly higher than THB group (Fig. 5A–B), similar to previous reports [7], [8]. However, by 90 and 150 min post CSP1 exposure, the amount of small DNA fragments and nucleotides released by the Δ*comA*+CSP1 became indistinguishable than the Δ*comA* (Fig. 5A–B).

**Figure 6:**
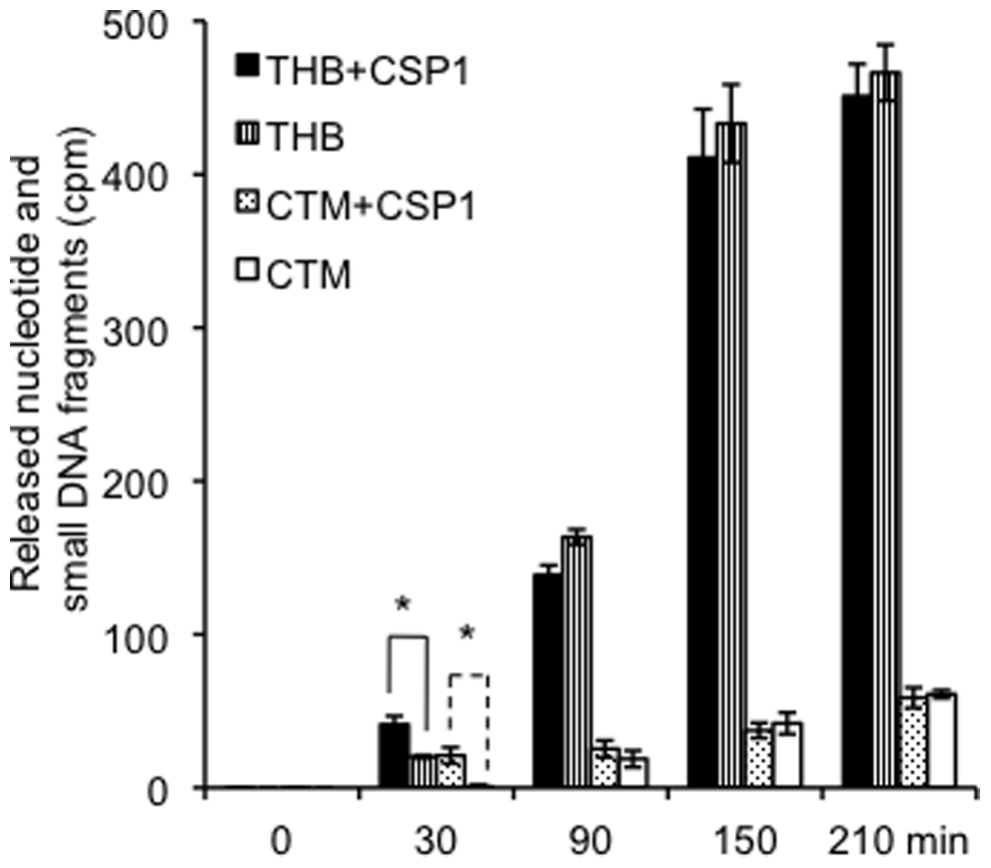
Quantitative detection of nucleotides and small DNA fragments released by pneumococcus with or without CSP1 stimulation in THB or CTM. Δ*comA* cells resuspended in fresh THB or CTM in the presence or absence of 400 ng/ml CSP1 were exposed to PCR-amplified P-32 labeled donor DNA (5 μ-3). The amount of nucleotides/small DNA fragments release at 0, 30, 90, 150 and 210 min after incubation were quantified by radiometric detection and expressed as counts per minute (cpm). The experiments were independently performed three times, in triplicates. The means ± SD of one representative experiment are shown. *p<0.05 when comparing the amount of released nucleotides and small DNA fragments of both THB and CTM grown *ΔcomA* supplemented with CSP1 against *ΔcomA* alone.

Because densitometry method only measures DNA degradation in a semi-quantitative manner, we repeated the experiments in Figure 5 using pneumococcal cells grown in THB and in CTM, and quantified the release of small DNA fragments and nucleotides by using a scintillation counter. As shown in Figure 6, after 30 min of incubation, nucleotides/small DNA fragments released by Δ*comA* grown in the CTM supplemented with CSP1 (21 cpm), is much higher than Δ*comA* grown in the CTM without CSP1 (0.73 cpm). Similarly, at this time interval, nucleotides/small DNA fragments released by Δ*comA* grown in the THB with CSP1 (41.23 cpm), is significantly higher than Δ*comA* grown in the THB without CSP1 (20.2 cpm). This indicates that the competence-induced DNA degradation is apparent at 30 min after CSP1 stimulation. After 90, 150 and 210 min of incubation, competence induced DNA degradation is no longer obvious. At these time intervals, nucleotides/small DNA fragments released by Δ*comA* grown in THB alone increase dramatically, and is statistically indistinguishable from Δ*comA* grown in THB supplemented with CSP1. In contrast, after 90, 150 and 210 min of incubation, small DNA fragments/nucleotide released by Δ*comA* grown in CTM was very low (Figure 6). These observations suggest that competence independent nuclease activity is low when Δ*comA* was cultured in CTM. Collectively, these results suggest the existence of both competence-dependent and competence-independent EndA nucleolytic activities. Competence-dependent EndA mediates rapid release of small DNA fragments right after CSP1 stimulation while competence-independent EndA activity mediates gradual release of small DNA fragments after the peak of competence for genetic transformation. Overall, the competence-dependent EndA activity is relatively weak and transient, and its contribution to DNA degradation is negligible under our experimental conditions.

### Secreted form of EndA Contributes Substantially to the “Competence-independent” Activity of EndA

**Figure 7:**
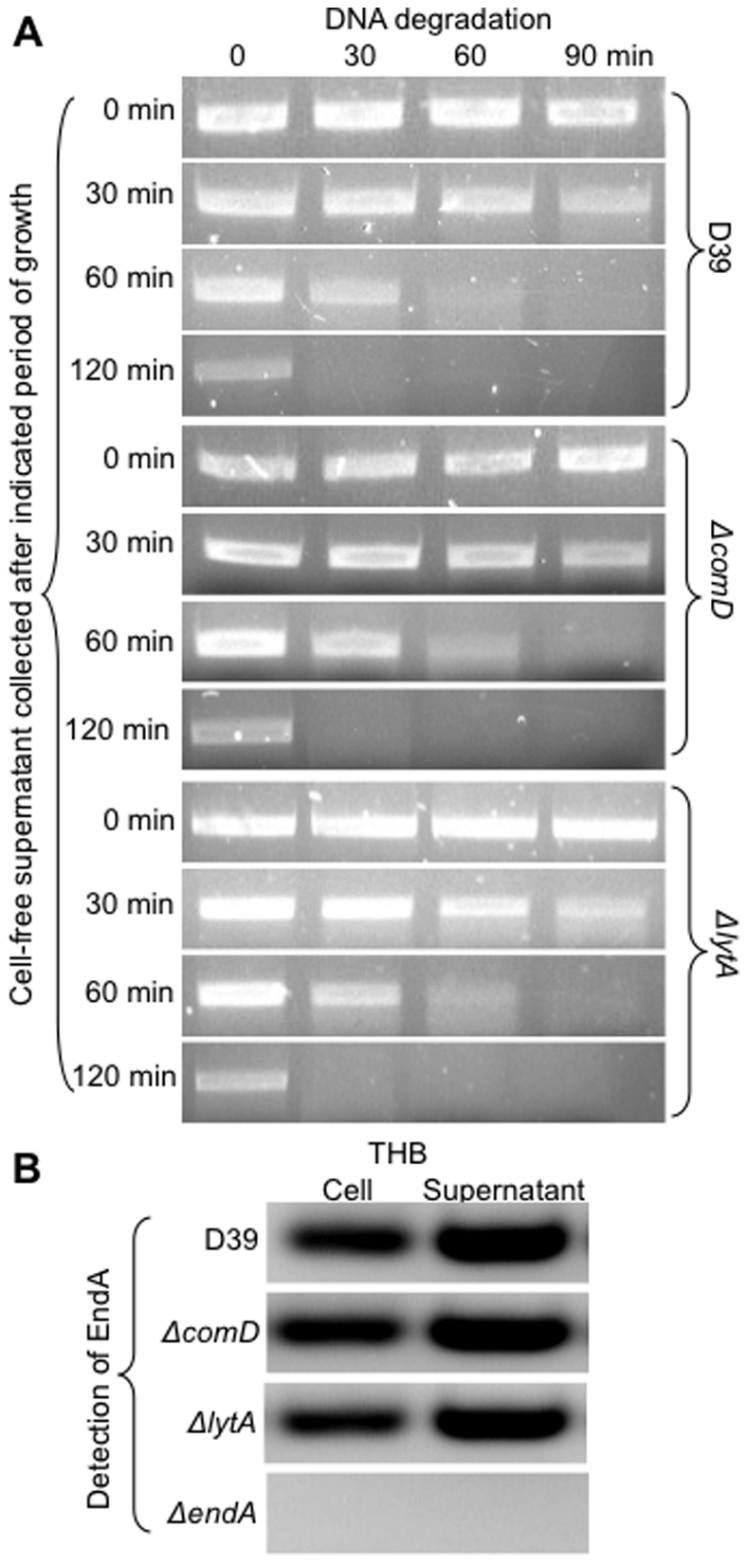
Competence-independent DNA degradation is caused by EndA secreted by pneumococcus into the culture medium. (A) The *rpsL* PCR products (30 µg/ml) were exposed to cell-free supernatants from D39 and its isogenic Δ*comD* and Δ*lytA* mutants collected during growth at the indicated time intervals. The integrity of DNA was visualized by agarose gel electrophoresis. (B) Bacterial cell lysates and cell-free supernatants from D39, Δ*comD* and Δ*lytA* cultured in THB were subjected to SDS-PAGE in a gel incorporated with 15 µg/ml of salmon sperm DNA. After renaturation, the gel was stained with ethidium bromide to visualize bands of DNA clearance by EndA. The experiments were performed independently three times. The results from one typical experiment are shown.

It has been purported that membrane-localized EndA, presumably recruited by the pseudopilus and other competence apparatus, can only gain access and degrade DNA when pneumococcal cells enter competent state (8). However, our experimental data suggest that the competence-independent activity of EndA is responsible for majority of DNA degradation. We hypothesized that pneumococcal cells secrete EndA into the culture medium during growth and contribute to the competence-independent degradation of the extracellular DNA. A small portion of the EndA molecules may have been recruited by the pseudopilus when pneumococcal cells are competent for genetic transformation. To determine whether EndA is secreted, cell-free supernatants of D39 and its isogenic, *ΔcomD* and *ΔlytA* mutants were collected overtime and assessed for their ability to degrade DNA. As shown in Fig. 7A, the nucleolytic activities increase in a time-dependent manner in the cell-free supernatants of D39, *ΔcomD* and *ΔlytA* grown in the THB. In addition, cell-free supernatants from *ΔcomD* and *ΔlytA* mutants degraded DNA to the same extent as D39, suggesting that competence development and cell lysis do not contribute significantly to the accumulation of secreted EndA.

To further confirm EndA is secreted into the culture medium, bacterial cells and cell-free supernatant were collected from D39 and its isogenic mutants *ΔcomD*, *ΔlytA*, and *ΔendA* and subjected to SDS-PAGE in a acrylamide gel incorporated with 15 µg/ml of salmon sperm DNA [5]. The gel was then washed with pure water to remove SDS and allow proteins to renature, and examined for DNA degradation after staining with ethidium bromide. As shown in Fig. 7B, EndA activities were detected in both bacterial lysate as well as cell-free supernatants from D39, *ΔcomD* and *ΔlytA*. In contrast, no nucleolytic band was visible in the *ΔendA*. These results strongly suggest that EndA is secreted in a process independent of competence development or partial cell lysis.

### EndA Secretion is Conserved in Different Pneumococcal Strains and is Culture Medium Dependent

**Figure 8:**
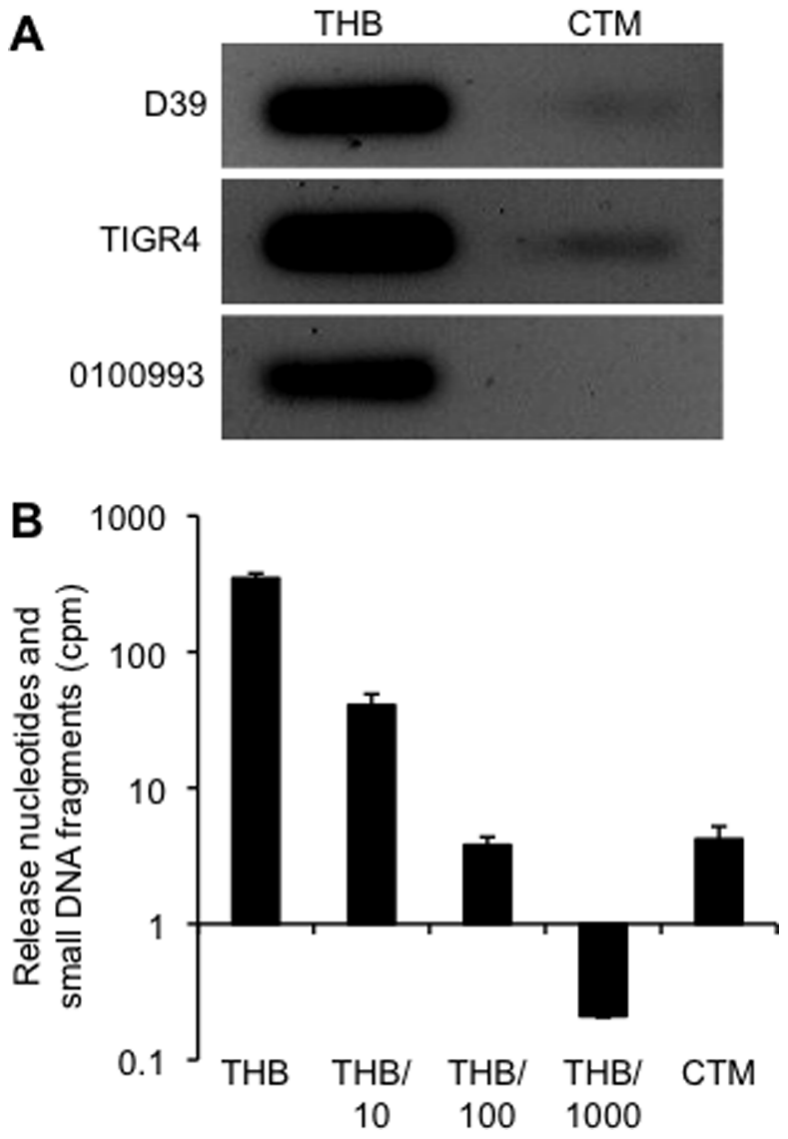
Pneumococcal strains grown in the CTM secrete lower amounts of EndA when compared to the same bacterial strains grown in the THB. EndA activity in the culture supernatant of D39 was determined and compared between pneumococcus growing in THB and CTM by in-gel digestion assay (A), and by quantitative DNA degradation assay in D39 cultured supernatant serially diluted with fresh THB (B). The experiments were performed independently in triplicates and repeated three times. The means ± SD of one representative experiment are shown.

Next, we compared the EndA secretion by various pneumococcal strains cultured in THB versus in CTM. D39, TIGR4 and 0100993 cultured in THB secreted much higher amounts of EndA when compared to these strains cultured in CTM (Figure 8A). To quantitatively compare the amount of EndA secretion by pneumococcus growing in THB versus in CTM, 1 ml of cell-free supernatant from D39 grown in THB were diluted 10 folds, 100 folds and 1000 folds. Nuclease activities of different diluents were compared against the nuclease activity of undiluted cell-free supernatant from D39 grown in CTM. Each supernatant samples were exposed to hot PCR product and incubated at 37C for 1 hr. The release of nucleotides/small DNA fragments was quantified by a scintillation counter. As shown in Fig. 8B, cell-free supernatant of D39 cultured in THB produced a nuclease activity of 348 cpm, decreasing to 40.6 cpm, 3.8 cpm and 0.21 cpm in 10 fold, 100 fold and 1000-fold THB diluents, respectively. In contrast, the nuclease activity of undiluted cell-free supernatant of D39 cultured in CTM is 4.2 cpm, which is approximates the 100-fold diluent of the THB supernatant. These results suggest that D39 cells growing in THB release about 100 times more EndA than D39 cells growing in CTM. These observations could partially explain weak nucleolytic activities when pneumococcal strains were cultured in CTM (Figs. 3–4).

### Degradation of NETs by EndA is Independent of Competence and is Inhibited by ATA

**Figure 9:**
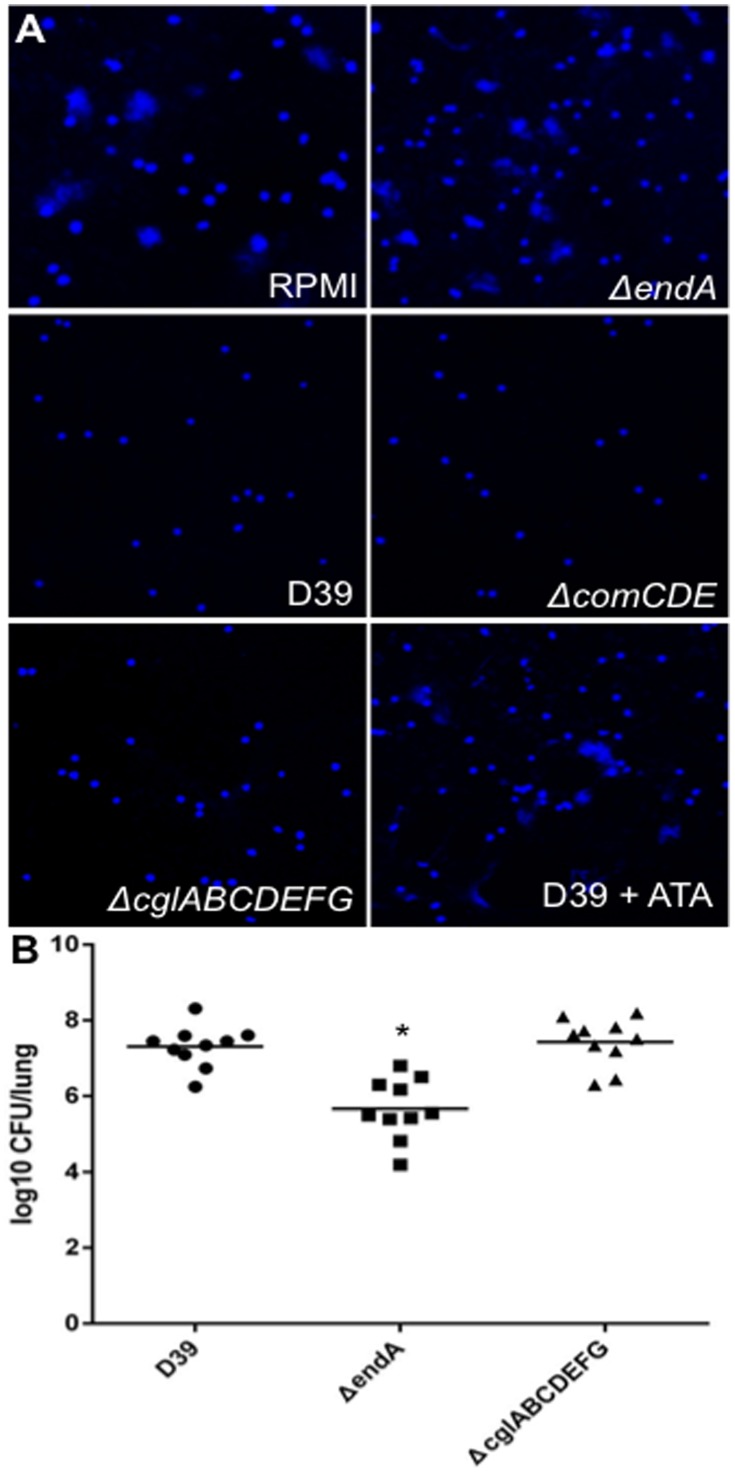
The competence-independent activity of EndA is important for virulence. (A) Degradation of NETs by EndA is independent of the development of competence. Neutrophils were activated to produce NETs before exposure to RPMI medium, wild-type D39 or mutant strains deficient in genetic transformation Δ*endA*, Δ*comCDE*, Δ*cglABCDEFG*, or D39+15 µg/ml ATA. The samples were stained with DAPI, and imaged under a fluorescence microscope. (B) Competence-dependent degradation of DNA by EndA is not important for lung infection. CD-1 mice (n = 10) were intranasally infected with 10^6^ of D39, Δ*endA* or Δ*cglABCDEFG*. Mouse lungs were harvested 48 hr post-infection for bacterial enumeration. * *p*<0.05 by GraphPad Prism statistical method when comparing bacterial load of Δ*endA* against D39 or Δ*cglABCDEFG*.

EndA has been previously shown to degrade NETs, a host defense mechanism elaborated by neutrophils [11]. We examined whether the EndA-mediated degradation of NETs in pneumococcus is dependent on the induction of the competence regulon. As expected, NETs were intact in RPMI culture medium or Δ*endA* treated neutrophils (Fig. 9A). In contrast, D39, Δ*comCDE* and Δ*cglABCDEFG* readily digested NETs (Fig. 9A). In addition, degradation of NETs by D39 cells was inhibited by 15 µg/ml ATA (Fig. 9A). Collectively, these results suggest that the degradation of NETs by EndA is independent of competence development.

### EndA but not CglABCDEFG is Required for Lung Infection

The DNA uptake apparatus of pneumococcus encoded by the *cglABCDEFG* operon was previously reported to be required for DNA degradation during competence [8]. To determine whether competent dependent-EndA-mediated DNA degradation is important for virulence, we compared the bacterial burden of D39, Δ*endA* and Δ*cglABCDEFG* using an acute pneumonia model of infection. The number of Δ*endA* bacteria was approximately 2 logs lower than that of the D39 in mouse lungs (n = 10). In contrast, the number of Δ*cglABCDEFG* bacteria was not significantly different from that of the D39 (Fig. 9B). This result suggests that the competence-independent activity of EndA is more important to the pneumococcal virulence than the EndA activities expressed when pneumococcus is competent for genetic transformation.

## Discussion

According to previous studies, nucleolytic activity of EndA was only detectable in pneumococcal cells competent for genetic transformation [7], [8]. Nucleolytic activity of EndA was also used as an indicator of competent state [7]. Here, we demonstrate the existence of competence-independent EndA activities that is responsible for the majority of the degradation of extracellular DNA and NETs, and is important for virulence. Several lines of experimental evidence support these conclusions: (i) the overall nucleolytic activity of competence-deficient mutants is comparable to their parental wild-type D39; (ii) addition of exogenous CSP1 that induces competence development contributes minimally to the overall rate of DNA degradation; (iii) partial cell autolysis, which may release EndA into DNA-rich environments, is not required for the nucleolytic activity; and (iv) secreted form of EndA contributes substantially to competence-independent nucleolytic activity of pneumococcus.

The aforementioned observations raise an intriguing question: why is it that in some studies [7], [8] the nucleolytic activity of EndA is only detectable during the competent state while we could detect nuclease activity in both competent and non-competent cells? We attribute part of these discrepancies to different strains used for the experiment. For example, the serotype III clinical isolate 0100993 has weaker nuclease activity than that of D39, R6 or TIGR4. This suggests that different pneumococcal strains express and/or secrete EndA at different levels. Another factor that determines EndA activities is the growth medium. We found that nucleolytic activity of EndA is severely reduced in the CTM, which is a nutritionally poorer medium when compared to the THB. CTM is a preferred medium used in many genetic transformation studies, likely because it allows the integrity of donor DNA to be preserved for a longer period of time. Based on this, we concluded that previous observation of competence-dependent EndA activity [7], [8] does not conflict with our observation of competence-independent EndA activity. Competence-dependent EndA activity is transient but detectable for a short period of time right after CSP1 stimulation. In contrast, competence-independent EndA activity predominates after longer incubation in a richer medium like THB, which favors EndA production.

EndA has been reported to be a transmembrane protein [5]. The thickness of D39 cell wall is estimated to be 23 nm [33]. However, according to the 3D modeling of protein structure using the VMD software program, estimated size of the EndA catalytic domain is approximately 4.5 nm (data not shown). It has been suggested that EndA could only gain access to extracellular DNA through the *cglABCDEFG* operon encoded pseudopilus uptake apparatus [8]. However, our results show that pneumococcal cells deficient in the Cgl proteins rapidly degrade extracellular DNA. A previous study has attributed the DNA degradation in non-competent pneumococcal cells to the release of EndA molecules into the medium by autolysis or other mechanisms [34]. However, our finding shows that the autolysis deficient Δ*lytA* mutant degrades DNA as rapidly as the parental wild-type D39. Furthermore, inclusion of choline chloride, which completely abolishes the function of all the autolysins including LytA, LytB, LytC and CbpD, neither delays nor attenuates DNA degradation. Collectively, these data refute the argument that rapid DNA degradation is caused by the release of EndA mediated by partial autolysis during pneumococcal growth. Rather, we provide strong evidence that EndA is secreted during normal growth of pneumococcus, which contributes substantially to the competence-independent DNA degradation.

In conclusion, we have shown that the main nucleolytic activity of pneumococcal EndA is independent of the competence development in THB. Competence-independent activity of EndA is responsible for rapid degradation of extracellular DNA, NETs, and is required for the virulence of *S. pneumoniae* during lung infection. Therefore, drugs that inhibit EndA, including ATA, could potentially be used to attenuate pneumococcal-mediated degradation of NETs and spread of infection, and reduce horizontal gene transfer. Also, because EndA is secreted and accessible by antibodies, it may serve as an attractive vaccine target.

## References

[1] Rosenthal AL, Lacks SA (1980) Complex structure of the membrane nuclease of *Streptococcus pneumoniae* revealed by two-dimensional electrophoresis. J Mol Biol 141: 133–146. 6255165 10.1016/0022-2836(80)90381-2

[2] Kohoutova DM (1961) Mechanism of the transformation of the polysaccharide capsule in Pneumococcus. Nature 190: 1171–1173. 13757619 10.1038/1901171a0

[3] Lacks S, Greenberg B (1973) Competence for deoxyribonucleic acid uptake and deoxyribonuclease action external to cells in the genetic transformation of *Diplococcus pneumoniae* . J Bacteriol 114: 152–163. 4144589 10.1128/jb.114.1.152-163.1973 PMC251751

[4] Lacks S, Greenberg B, Neuberger M (1974) Role of a deoxyribonuclease in the genetic transformation of *Diplococcus pneumoniae* . Proc Natl Acad Sci U S A 71: 2305–2309. 4152205 10.1073/pnas.71.6.2305 PMC388441

[5] Lacks S, Greenberg B, Neuberger M (1975) Identification of a deoxyribonuclease implicated in genetic transformation of *Diplococcus pneumoniae* . J Bacteriol 123: 222–232. 237879 10.1128/jb.123.1.222-232.1975 PMC235710

[6] Lacks S (1962) Molecular fate of DNA in genetic transformation of Pneumococcus. J Mol Biol 5: 119–131. 14461406 10.1016/s0022-2836(62)80067-9

[7] Chen JD, Morrison DA (1987) Modulation of competence for genetic transformation in *Streptococcus pneumoniae* . J Gen Microbiol 133: 1959–1967. 3668504 10.1099/00221287-133-7-1959

[8] Berge M, Moscoso M, Prudhomme M, Martin B, Claverys JP (2002) Uptake of transforming DNA in Gram-positive bacteria: a view from *Streptococcus pneumoniae* . Mol Microbiol 45: 411–421. 12123453 10.1046/j.1365-2958.2002.03013.x

[9] Hava DL, Camilli A (2002) Large-scale identification of serotype 4 *Streptococcus pneumoniae* virulence factors. Mol Microbiol 45: 1389–1406. 12207705 PMC2788772

[10] Beiter K, Wartha F, Albiger B, Normark S, Zychlinsky A, et al (2006) An endonuclease allows *Streptococcus pneumoniae* to escape from neutrophil extracellular traps. Curr Biol 16: 401–407. 16488875 10.1016/j.cub.2006.01.056

[11] Brinkmann V, Reichard U, Goosmann C, Fauler B, Uhlemann Y, et al (2004) Neutrophil extracellular traps kill bacteria. Science 303: 1532–1535. 15001782 10.1126/science.1092385

[12] Avery OT, MacLeod CM, McCarty M (1979) Studies on the chemical nature of the substance inducing transformation of pneumococcal types. Inductions of transformation by a desoxyribonucleic acid fraction isolated from pneumococcus type III. J Exp Med 149: 297–326. 33226 10.1084/jem.149.2.297 PMC2184805

[13] Avery OT, Macleod CM, McCarty M (1944) Studies on the Chemical Nature of the Substance Inducing Transformation of Pneumococcal Types : Induction of Transformation by a Desoxyribonucleic Acid Fraction Isolated from Pneumococcus Type Iii. J Exp Med 79: 137–158. 19871359 10.1084/jem.79.2.137 PMC2135445

[14] Lau GW, Haataja S, Lonetto M, Kensit SE, Marra A, et al (2001) A functional genomic analysis of type 3 *Streptococcus pneumoniae* virulence. Mol Microbiol 40: 555–571. 11359563 10.1046/j.1365-2958.2001.02335.x

[15] Sung CK, Li H, Claverys JP, Morrison DA (2001) An rpsL cassette, janus, for gene replacement through negative selection in *Streptococcus pneumoniae* . Appl Environ Microbiol 67: 5190–5196. 11679344 10.1128/AEM.67.11.5190-5196.2001 PMC93289

[16] Sung CK, Morrison DA (2005) Two distinct functions of ComW in stabilization and activation of the alternative sigma factor ComX in *Streptococcus pneumoniae* . J Bacteriol 187: 3052–3061. 15838032 10.1128/JB.187.9.3052-3061.2005 PMC1082825

[17] Zhu L, Lau GW (2011) Inhibition of competence development, horizontal gene transfer and virulence in *Streptococcus pneumoniae* by a modified competence stimulating peptide. PLoS Pathog 7: e1002241. 21909280 10.1371/journal.ppat.1002241 PMC3164649

[18] Morrison DA, Trombe MC, Hayden MK, Waszak GA, Chen JD (1984) Isolation of transformation-deficient *Streptococcus pneumoniae* mutants defective in control of competence, using insertion-duplication mutagenesis with the erythromycin resistance determinant of pAM beta 1. J Bacteriol 159: 870–876. 6090396 10.1128/jb.159.3.870-876.1984 PMC215739

[19] Jeffries CD, Holtman DF, Guse DG (1957) Rapid method for determining the activity of microorganisms on nucleic acids. J Bacteriol 73: 590–591. 13428699 10.1128/jb.73.4.590-591.1957 PMC314626

[20] Johnsborg O, Kristiansen PE, Blomqvist T, Havarstein LS (2006) A hydrophobic patch in the competence-stimulating Peptide, a pneumococcal competence pheromone, is essential for specificity and biological activity. J Bacteriol 188: 1744–1749. 16484185 10.1128/JB.188.5.1744-1749.2006 PMC1426553

[21] Liu CL, Tangsombatvisit S, Rosenberg JM, Mandelbaum G, Gillespie EC, et al (2012) Specific post-translational histone modifications of neutrophil extracellular traps as immunogens and potential targets of lupus autoantibodies. Arthritis Res Ther 14: R25. 22300536 10.1186/ar3707 PMC3392818

[22] Hallick RB, Chelm BK, Gray PW, Orozco EM Jr (1977) Use of aurintricarboxylic acid as an inhibitor of nucleases during nucleic acid isolation. Nucleic Acids Res 4: 3055–3064. 410006 10.1093/nar/4.9.3055 PMC342634

[23] Pestova EV, Havarstein LS, Morrison DA (1996) Regulation of competence for genetic transformation in *Streptococcus pneumoniae* by an auto-induced peptide pheromone and a two-component regulatory system. Mol Microbiol 21: 853–862. 8878046 10.1046/j.1365-2958.1996.501417.x

[24] Mellroth P, Daniels R, Eberhardt A, Ronnlund D, Blom H, et al. (2012) LytA, the major autolysin of *Streptococcus pneumoniae*, requires access to the nascent peptidoglycan. J Biol Chem. 10.1074/jbc.M111.318584 PMC3322828 22334685

[25] Garcia P, Gonzalez MP, Garcia E, Lopez R, Garcia JL (1999) LytB, a novel pneumococcal murein hydrolase essential for cell separation. Mol Microbiol 31: 1275–1281. 10096093 10.1046/j.1365-2958.1999.01238.x

[26] Briese T, Hakenbeck R (1985) Interaction of the pneumococcal amidase with lipoteichoic acid and choline. Eur J Biochem 146: 417–427. 3967665 10.1111/j.1432-1033.1985.tb08668.x

[27] Mellroth P, Daniels R, Eberhardt A, Ronnlund D, Blom H, et al (2012) LytA, major autolysin of *Streptococcus pneumoniae*, requires access to nascent peptidoglycan. J Biol Chem 287: 11018–11029. 22334685 10.1074/jbc.M111.318584 PMC3322828

[28] Steinmoen H, Knutsen E, Havarstein LS (2002) Induction of natural competence in *Streptococcus pneumoniae* triggers lysis and DNA release from a subfraction of the cell population. Proc Natl Acad Sci U S A 99: 7681–7686. 12032343 10.1073/pnas.112464599 PMC124321

[29] Moscoso M, Garcia E, Lopez R (2006) Biofilm formation by *Streptococcus pneumoniae*: role of choline, extracellular DNA, and capsular polysaccharide in microbial accretion. J Bacteriol 188: 7785–7795. 16936041 10.1128/JB.00673-06 PMC1636320

[30] Luo P, Li H, Morrison DA (2003) ComX is a unique link between multiple quorum sensing outputs and competence in *Streptococcus pneumoniae* . Mol Microbiol 50: 623–633. 14617184 10.1046/j.1365-2958.2003.03714.x

[31] Lee MS, Morrison DA (1999) Identification of a new regulator in *Streptococcus pneumoniae* linking quorum sensing to competence for genetic transformation. J Bacteriol 181: 5004–5016. 10438773 10.1128/jb.181.16.5004-5016.1999 PMC93990

[32] Alloing G, Martin B, Granadel C, Claverys JP (1998) Development of competence in *Streptococcus pneumonaie*: pheromone autoinduction and control of quorum sensing by the oligopeptide permease. Mol Microbiol 29: 75–83.33. Chen I, Dubnau D (2004) DNA uptake during bacterial transformation. Nat Rev Microbiol 2: 241–249. 10.1046/j.1365-2958.1998.00904.x 9701804

[33] Tran TD, Kwon HY, Kim EH, Kim KW, Briles DE, et al (2011) Decrease in penicillin susceptibility due to heat shock protein ClpL in *Streptococcus pneumoniae* . Antimicrob Agents Chemother 55: 2714–2728. 21422206 10.1128/AAC.01383-10 PMC3101445

[34] Moscoso M, Claverys JP (2004) Release of DNA into the medium by competent *Streptococcus pneumoniae*: kinetics, mechanism and stability of the liberated DNA. Mol Microbiol 54: 783–794. 15491367 10.1111/j.1365-2958.2004.04305.x

[35] Tettelin H, Nelson KE, Paulsen IT, Eisen JA, Read TD, et al (2001) Complete genome sequence of a virulent isolate of *Streptococcus pneumoniae* . Science 293: 498–506. 11463916 10.1126/science.1061217

