# Correction: Competence-Independent Activity of Pneumococcal Enda Mediates Degradation of Extracellular DNA and Nets and Is Important for Virulence

**DOI:** 10.1371/annotation/75f51a45-a2de-4893-94cf-0045056a4d7c

**Published:** 2013-09-23

**Authors:** Luchang Zhu, Zhizhou Kuang, Brenda A. Wilson, Gee W. Lau

There were multiple capitalization errors in the article title.

The correct title is: "Competence-Independent Activity of Pneumococcal EndA Mediates Degradation of Extracellular DNA and NETs and is Important for Virulence".

The correct citation is: Zhu L, Kuang Z, Wilson BA, Lau GW (2013) Competence-Independent Activity of Pneumococcal EndA Mediates Degradation of Extracellular DNA and NETs and is Important for Virulence. PLoS ONE 8(7): e70363. doi:10.1371/journal.pone.0070363 

